# Combined Effects of Color and Elastic Modulus on Antifouling Performance: A Study of Graphene Oxide/Silicone Rubber Composite Membranes

**DOI:** 10.3390/ma12162608

**Published:** 2019-08-16

**Authors:** Huichao Jin, Wei Bing, Limei Tian, Peng Wang, Jie Zhao

**Affiliations:** 1 Key Laboratory of Bionic Engineering, Ministry of Education, Jilin University, Changchun 130022, China; 2 School of Mechanical Engineering and Automation, Beihang University, Beijing 100191, China; 3 College of Physics, Jilin University, Changchun 130012, China; 4 Advanced Institute of Materials Science, Changchun University of Technology, Changchun 130012, China; 5 State Key Laboratory of Superhard Materials, Jilin University, Changchun 130012, China

**Keywords:** graphene oxide, silicone rubber, composite materials, antifouling, harmonic motion

## Abstract

Biofouling is a significant maritime problem because the growth of fouling organisms on the hulls of ships leads to very high economic losses every year. Inspired by the soft skins of dolphins, we prepared graphene oxide/silicone rubber composite membranes in this study. These membranes have low surface free energies and adjustable elastic moduli, which are beneficial for preventing biofouling. Diatom attachment studies under static conditions revealed that color has no effect on antifouling behavior, whereas the studies under hydrodynamic conditions revealed that the combined effects of color and elastic modulus determine the antifouling performance. The experimental results are in accordance with the “harmonic motion effect” theory proposed by us, and we also provide a supplement to the theory in this paper. On the basis of the diatom attachment test results, the membrane with 0.36 wt % of graphene oxide showed excellent antifouling performance, and is promising in practical applications. The results confirmed that the graphene oxide and graphene have similar effect to enhance silicone rubber antifouling performance. This study provides important insight for the design of new antifouling coatings; specifically, it indicates that lighter colors and low Young’s moduli provide superior performance. In addition, this study provides a reference for the application of graphene oxide as fillers to enhance the composite antifouling performance.

## 1. Introduction

Biofouling is a significant maritime problem because fouling organisms that grow on the hulls of ships promote their deterioration and increase drag, thereby increasing fuel costs [1,2]. Another impact of biofouling is bioinvasion [3]. In the coast of California, more than 60% of invasive species arrived by clinging to the surface of ships [4]. To solve these problems, the California government announced regular biological inspection of hulls starting from January 2018 to reduce the invasion of alien species [5]. These negative impacts have caused enormous economic losses worldwide [6]. Intense methods have been developed to combat biofouling, including the use of copper coatings [7] and tributyltin self-polishing copolymer (TBT-SPC) [8]. However, in the 1980s, a series of studies reported the high toxicity of TBT-SPC to marine organisms [9,10]. Since then, the use of toxic antifouling coatings has gradually been banned in many countries [11,12]. Therefore, the development of new eco-friendly antifouling coatings is urgent.

In recent years, composite coatings have aroused considerable interest as economic and eco-friendly solutions for preventing marine biofouling [13]. Composite coatings based on polydimethylsiloxane, silicone, and polyurethane-acrylate, among others, have been shown to prevent biofouling effectively [14,15,16]. Recently, considerable effort has been devoted to developing new composite antifouling coatings with graphene/graphene oxide (GO) as fillers [17,18], which promote the antifouling properties of the coatings [19]. However, several challenges still need to be overcome before these methods can be applied, such as high cost and low durability [20]. Hence, low cost and efficient antifouling coatings need to be designed and developed.

Dolphins, soft corals (*Sarcophyton trocheliophorum*), and seals were found to have antifouling capacity. These organisms have elastic skin, enabling the creation of a dynamic surface around the flow to resist biofouling, which is called the “harmonic motion effect.” [21,22] Inspired by this antifouling strategy, six GO/silicone rubber (GOSR) composite membranes were prepared in the present study. The preparation method is simple, highly efficient, and low-cost, and the resulting GOSR membranes are environmentally friendly. They are characterized by adjustable Young’s moduli, low surface free energies, and smooth surfaces, which are conducive to preventing biofouling. In this study, diatoms were selected as fouling organisms. The diatom attachment was examined under both static and hydrodynamic conditions, which revealed that color and Young’s modulus play major roles in diatom attachment. We previously reported a new understanding of the effect of elastic modulus on antifouling performance, which we referred to as the “harmonic motion effect” [21]; the experimental results of the present study can be explained on the basis of this effect. This study provides insight that will be useful for the design and fabrication of new antifouling coatings.

## 2. Materials and Methods

### 2.1. Materials

Silicone rubber (SR) was purchased from Guangdong Bo Rui Co., Ltd, Shenzhen, China. GO was purchased from Yuhuang New Energy Technology Co., Ltd, Heze, China. Acetone, tetrahydrofuran, and anhydrous ethanol were supplied by Beijing Chemical Works (Beijing, China). The diatom *Triceratium* sp. was obtained from Nanhuaqianmu Biotechnology Co. Ltd, Zhengzhou, China. Algal broth medium (028820) was purchased from Huankai Microbial Sci. & Tech. Co., Ltd, Guangzhou, China.

### 2.2. Membrane Preparation 

**Scheme 1:**
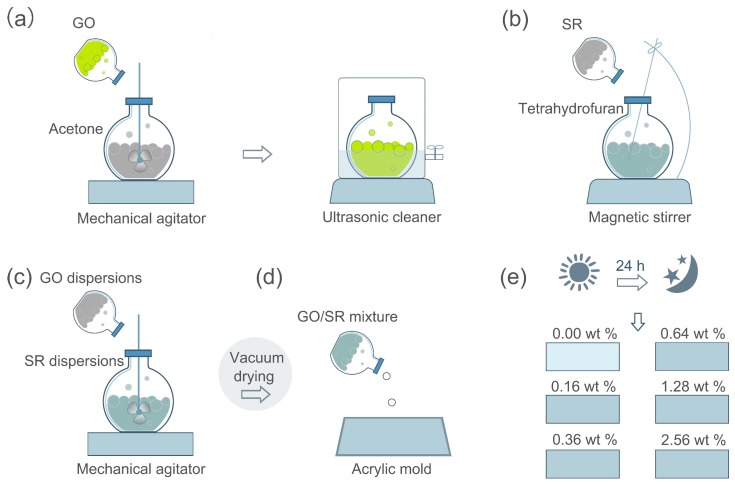
Preparation schematic of graphene/graphene oxide (GO)/silicone rubber (GOSR) composite membranes: (**a**) Preparation of a GO dispersion, (**b**) preparation of an silicone rubber (SR) dispersion, (**c**) the two dispersions are mixed to produce GOSR composite material, and (**d**,**e**) the mixture is poured into an acrylic mold and cured.

Scheme 1 illustrates the preparation of the GOSR composite membranes. (a) First, GO was added to acetone under mechanical agitation for 3 h, then remove the mixture into an ultrasonic cleaner for 3 h, which yielded GO dispersions, and the weight ratio of GO/acetone was 1/9. (b) SR was added to tetrahydrofuran under magnetic stirrer for 2 h, then SR dispersions were obtained, and the weight ratio of SR/tetrahydrofuran was 1/4. (c) According to the following Equation,

(1) $$\mathrm{Weight}\;\mathrm{percent}\;(\mathrm{wt}\%)\;=\;\frac{1/10M_{\mathrm{Go}\;\mathrm{dispersions}}}{1/10M_{\mathrm{Go}\;\mathrm{dispersions}}\;+\;1/5M_{\mathrm{SR}\;\mathrm{dispersions}}}$$

Five different GO/SR composite materials were prepared, the GO in composite materials were expressed as 0.16, 0.36, 0.64, 1.28, and 2.56 wt %, respectively. The mixtures were then stirred for 1 h under mechanical agitation, after which they were placed in a vacuum chamber for 1 h at 60 °C until acetone and tetrahydrofuran were removed. (d–e) Finally, the mixtures were poured into an acrylic mold and cured at room temperature for 24 h. A pristine SR (PSR) membrane with 0 wt % GO was prepared as a control.

### 2.3. Diatom Attachment Testing

First, 3.8 g of the algal broth medium was added to 1 L of deionized water, and the mixture was heated in an autoclave for 15 min at 121 °C. After cooling, 100 g of diatoms was added, and the mixture was cultured for 2 days at room temperature. As diatoms are photosynthetic organisms, a light-emitting diode (LED) plant lamp was used with a light/dark time-cycle of 14/10 h per day. After culturing for 2 days (see Figure S1, Supporting Information), the diatom suspension was added to containers for static and hydrodynamic diatom attachment tests. The containers were filled with 50 L of water and 50 g of the algal broth medium. The size of the specimens was approximately 5 cm × 2 cm × 2 mm (length × width × thickness). The specimens were submerged in a static container for 8 days, after which they were examined and measured. In the hydrodynamic test, the specimens were fixed to a hexagonal prism, and the speed of the electric motor was set as 500 rad/min. Therefore, the linear velocity (*V*) near the specimens was 3.4 m/s (Figure S2 and Table S1). Some studies reported the negative relevant relations between the amount of fouling organisms adhered on ship hulls and the flow velocity [23,24]. Therefore, a low flow velocity can reveal the antifouling capability of coatings under hydrodynamic conditions. The specimens were examined after 10 days.

### 2.4. Characterization

Raman spectra was obtained using a Raman spectrometer (T64000, HORIBA, Paris, France) which combined an Olympus microscope lens with a 532.05 nm CW He-Ne laser. The composition of GO nanosheets were analyzed using a Fourier transform infrared spectroscopy (FTIR, Thermo Fisher Scientific, Nicolet 6700, Waltham, MA, USA). The morphologies of GO nanosheets were obtained using a transmission electron microscope (TEM, JEOL, JEM1200EX, Mitaka-shi, Japan). The elemental composition was analyzed using an energy dispersive spectrometer (EDS, X-Max50, Oxford, UK) installed in a scanning electron microscope (JSM-7610, JEOL, Mitaka-shi, Japan). The water contact angles were measured using a surface tension meter (WCA, XG-CAM, Xuanyichuangxi, Shanghai, China) at room temperature with deionized water as testing liquid, and three specimens were measured for each membrane. The Young’s moduli were tested using a rubber testing machine (UTM5305, YOUHONG, Shanghai, China), and three specimens were measured for each membrane. The surface topographies of the specimens were analyzed by scanning probe microscopy (SPM, ICON, BRUKER, Karlsruhe, Germany). Specimens under static and hydrodynamic conditions were observed by scanning electron microscopy (SEM; MAGELLAN 400, FEI, Hillsboro, Oregon, USA, and JSM-7610, JEOL, Mitaka-shi, Japan). After the diatom attachment test, the six specimens were removed and placed into the same amounts of normal saline (4 ml), after which they were placed in an ultrasonic cleaner for 1 h to separate the diatoms from the specimens, and to produce diatom solutions. The optical densities of these solutions were measured at 440 nm (OD_440_) using an ultraviolet-visible spectrophotometer (UV-5500/PC, METASH, Shanghai, China), which revealed the amounts of diatoms on the different specimen surfaces. Three specimens were measured for each membrane to minimize the experimental error.

## 3. Results and Discussions

### 3.1. Membrane Composition Analysis

**Figure 1:**
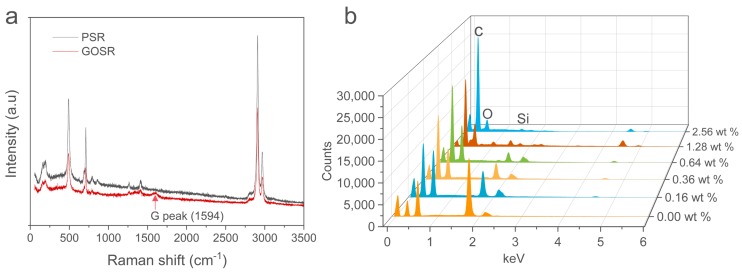
Characterization of specimens with different GO contents: (**a**) Raman spectra, (**b**) energy dispersive spectrometer (EDS) results.

The morphologies of GO nanosheets were observed by SEM (Figure S3a) and TEM (Figure S3b). As can be seen, the GO nanosheets resemble crumpled silk veil waves, which indicates that they are multi-layered. The GO nanosheets were confirmed by Raman spectroscopy (Figure S3c), and it is evident that GO has typical D (1359 cm^−1^) and G (1589 cm^−1^) peaks and a 2D region [25,26]. The functional groups of GO nanosheets were analyzed by FTIR (Figure S3d). The band at 3199.55 cm^−1^ is assigned to the O−H stretching vibration. The peak at 1722.31 cm^−1^ is attributed to C=O stretching vibration. The band at 1617.94 cm^-1^ is assigned to C=C skeletal vibration. The three peaks at 1401.60 cm^−1^, 1221.08 cm^−1^, and 1049.12 cm^−1^ correspond to the C−O stretching vibration. The Raman spectra of the GOSR membrane (red curve in Figure 1a) exhibits a peak at 1594 cm^−1^, which is the G peak of GO. The elemental compositions of the membranes were determined using the EDS (Figure 1b), and the results in Table S2 reveal that the carbon content increases with increasing GO content. The presence of GO in the membranes is evident (Figure S4). The PSR membrane appears milky white and semi-transparent, whereas the GOSR membranes are brown because of the imbedded GO, and the color deepens with increasing GO content. 

### 3.2. Membrane Properties

**Figure 2:**
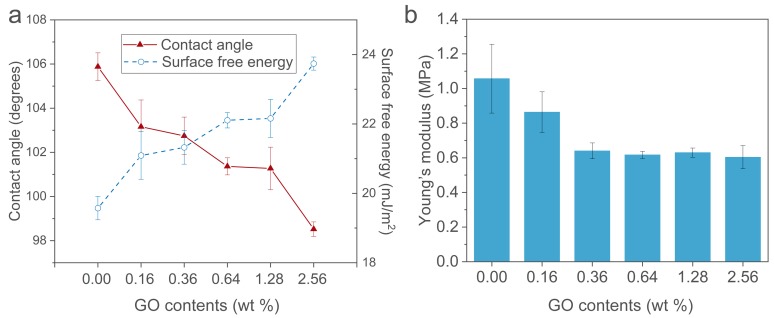
Characterization of specimens with different GO contents: (**a**) Water contact angles and surface free energies, (**b**) Young’s moduli.

The surface free energies, Young’s moduli, and surface topographies of these membranes were also examined, as they are vital for bio-adhesion. The contact angles of GOSR were measured (Figure 2a, red curve), and the correlation between contact angle and surface energy is given by [27]:

(2) $$\cos\theta \;=\;-1\;+\;2\sqrt{\frac{\gamma_{S}}{\gamma_{L}}}\;[1-\beta (\gamma_{L}-\gamma_{S}{)}^{2}]$$

where *θ* stands for contact angle, *γ_S_* and *γ_L_* represents surface energy of solid and liquid, respectively. *β* is a constant with the value 1.057 × 10^−4^ m^2^/mJ, and surface energy of deionized water is 72.8 mJ/m^2^.

**Figure 3:**
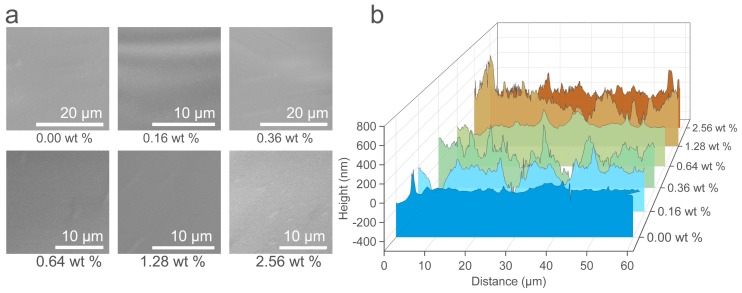
(**a**) SEM images of pristine SR (PSR)/GOSR membrane surfaces. (**b**) Scanning probe microscopy (SPM) height profiles of PSR/GOSR membranes.

The surface free energies of the membranes with varying GO contents range from 19.57 mJ·m^−2^ to 23.74 mJ·m^−2^ (Figure 2a, blue curve), which are conducive to reducing marine biofouling [28]. It is clear that GO significantly affects the Young’s modulus (Figure 2b). Some studies have reported that Young’s modulus increases with GO contents [29]. However, the Young’s modulus results from this test contradict reported data as well as our previous studies [21,30], which is ascribable to the GO not being pre-treated with the coupling agent (KH-550) in this experiment. The GO in the membrane can aggregate, resulting in points of converging stress in the membrane [17]; consequently, the Young’s modulus decreases with increasing GO contents. Another explanation is that an excess of the GO filler will weaken the interaction between the polymer chain segments and decrease the tensile strength [31]. It is generally assumed that a low Young’s modulus is beneficial for mitigating the adhesion of fouling organisms [21,32]. The surface topographies were also examined. Figure 3a reveals that the membranes have smooth surfaces at the micron level, and this low surface roughness is important for combating biofouling [33]. In addition, SPM results (Figure 3b and Figure S5) show that nanostructures are present on the surfaces, and some studies reported that nanoscale roughness is conducive to combating biofouling [34].

### 3.3. Diatom Adhesion Testing Under Static Conditions

**Figure 4:**
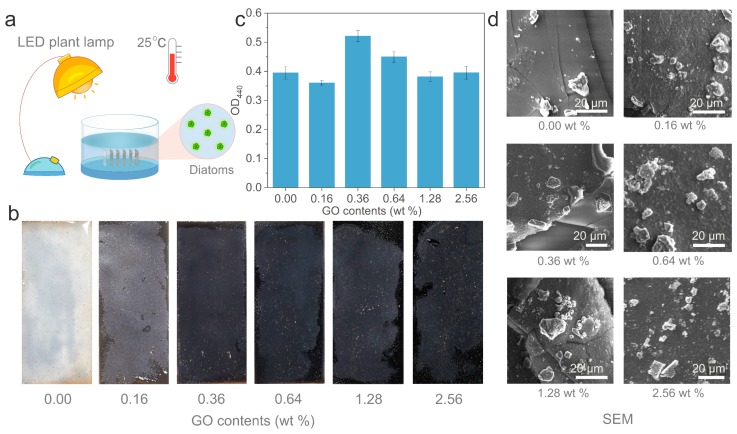
(**a**) Schematic illustration of diatom adhesion testing under static conditions. (**b**) Optical images of sample surfaces at 0 days and 8 days. (**c**) OD_440_ results of the membranes after 8 days. (**d**) SEM images of specimens after 8 days.

The specimens were examined after soaking for 8 days. In summary, these specimens show similar results (Figure 4b,c), and no biofilms were observed (Figure 4d), and only scattered diatoms were present on the surfaces. It is commonly assumed that the Young’s modulus does not influence static attachment [35]. Since the surface free energies of the specimens are similar (Figure 2a), they show similar diatom attachment results which conform to the prediction of "Baier curve" [28]. Previous studies confirmed that diatoms prefer to adhere to dark surfaces [36,37]. However, their experiments were performed under hydrodynamic conditions. The results in Figure 4 show that colors have no effect on diatom adhesion under static conditions.

### 3.4. Diatom Adhesion Testing Under Hydrodynamic Conditions

**Figure 5:**
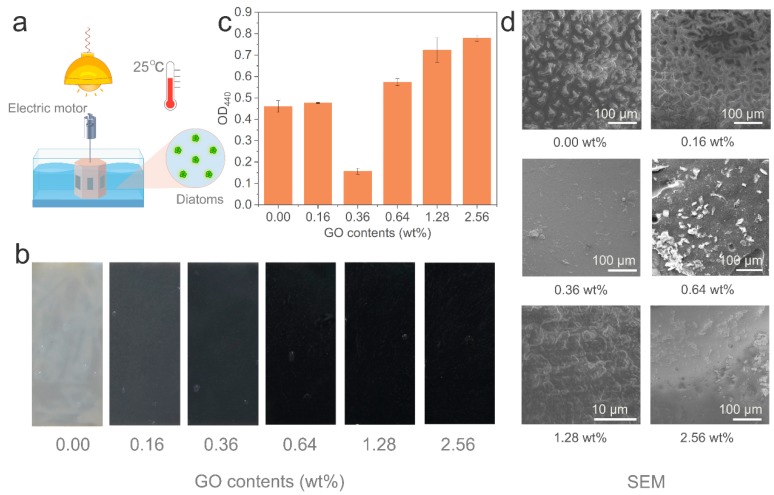
(**a**) Schematic illustration of diatom adhesion testing under hydrodynamic conditions. (**b**) Optical images of sample surfaces at 0 days and 10 days. (**c**) OD_440_ results of the membranes after 10 days. (**d**) SEM images of specimens after 10 days.

The specimens were examined after 10 days. Biofilms were observed on the 0 wt %, 0.16 wt %, and 1.28 wt % membranes (Figure 5d). The 0.36 wt % membrane showed the cleanest surface (Figure 5c,d), and scattered diatoms were present on the 0.64 wt % and 2.56 wt % membranes. A low Young’s modulus is beneficial to combat adhesion of fouling organisms [21,38]. According to Figure 2b and Figure 5, the 0.64 wt %, 1.28 wt %, and 2.56 wt % GO-containing membranes have the lowest Young’s moduli; however, they show poor antifouling performances, due to their dark colors. On the other hand, the 0 wt % and 0.16 wt % membranes perform poorly due to high Young’s moduli. The 0.36 wt % membrane with appropriate color and Young’s moduli exhibits the best antifouling performance. We conclude that the combined effects of color and Young’s modulus determine the antifouling performance of GOSR membranes. 

**Figure 6:**
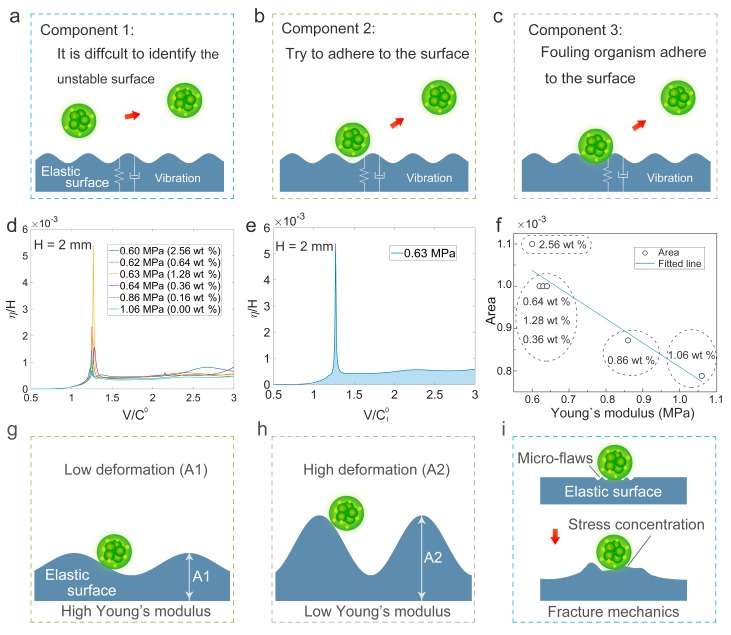
(**a**–**c**) Contributions of “harmonic motion” to biofouling resistance. (**d**) Deformations (*η/H*) of elastic membranes as functions of *V/*$C_{t}^{0}$ for different Young’s moduli. (**e**) *E* = 0.63 MPa curve. (**f**) Area as a function of Young’s modulus. (**g**) Elastic surface with high Young’s modulus. (**h**) Elastic surface with low Young’s modulus. (**i**) Schematic illustration of the fracture mechanics on antifouling.

In our previous studies, we proposed the “harmonic motion effect” to explain the antifouling behavior of elastic membranes [21], in which the deformation of an elastic membrane under turbulent flow imparts biofouling resistance. Biofouling resistance involves three components: (i) Fouling organisms have difficulty identifying dynamic surfaces (Figure 6a); (ii) when these organisms approach an elastic membrane, its dynamic surface sweeps them away (Figure 6b); (iii) if some organisms adhere to the elastic membrane (Figure 6c), they cannot adhere tightly because of the micro-flaws [21] produced at the interface. 

To reveal the mechanism via which a low elastic modulus aids in combating fouling organisms in the present study, a theory by Kulik is introduced below. Studies by Kulik revealed that the deformation amplitude of the elastic coatings in turbulent flow is approximately 0 μm to a few microns [39,40]. Three equations [41] (from Kulik) were employed to calculate the deformation amplitude of a surface:

(3) $$C_{n}^{*}\;=\;\frac{C_{n}}{H/E}\;=\;\frac{\lambda }{H}\;(\frac{V}{C_{t}^{0}})2^{\;}\frac{\left(1+\sigma \right)\alpha F}{2\pi {\left(1-i\mu \right)}^{2}\left[2-\;\frac{{\left(V/C_{t}^{0}\right)}^{2}}{1-i\mu }\;-\;2S\right]}$$

(4) $$\eta_{\mathrm{rms}}=\sqrt{\bar{\eta^{2}}}=[\int_{0}^{\infty }\;{\left|C_{n}\left(\omega \right)\right|}^{2}P\left(\omega \right)d\omega {]}^{1/2},$$

where $C_{n}^{*}$ is the vertical (normal to the surface) compliance; *P(ω)* stands for the energy spectrum of pressure pulsations; *η_rms_* denotes the vertical displacement; *η* and *H* are deformation and elastic-membrane thickness, respectively; and *V* and $C_{t}^{0}$ are the flow rate and shear wave rate for an ideal material, respectively.

*η/H* is plotted as a function of *V/*$C_{t}^{0}$ for various Young’s moduli in Figure 6d. In a practical situation, the flow velocity is not constant; thus, various velocities need to be studied. Figure 6e shows the curve of *E* = 0.63 MPa; the area between the curve and the x-axis (blue area in Figure 6e) characterizes the total deformation of membrane surface. Figure 6f shows the areas for *E* = 0.60−1.06 MPa (Figure 2b), which reveal that a lower Young’s modulus leads to a bigger area, i.e., a bigger deformation. According to contact mechanics [20,42], this higher deformation is conducive to combating the adhesion of fouling organisms (Figure 6g,h). Thus, 0 wt % and 0.16 wt % membranes show poor antifouling performances, due to their high Young’s modulus. On the basis of this theory, the 0.64 wt %, 1.28 wt %, and 2.56 wt % GO-containing membranes have a low Young’s moduli which are conducive to resisting biofouling; however, they show poor antifouling performances, due to their dark colors. In the past, fracture mechanics [38,43] was employed to reveal the mechanisms of fouling organisms and elastic surfaces. Figure 6i shows a schematic illustration of the fracture mechanics, which indicates that the contact between the fouling organism and elastic surface is not strong enough; consequently, there are some microcracks at the contact area. Fouling organism tend to detach from the surface when the microcracks grow increasing large due to the stress concentration. The Griffith theory of brittle fracture is as follows [43]:

(5) $$F=\sqrt{\frac{2E\gamma }{A\pi }}$$

where *F* represents the stress; A stands for the crack length; *E* represents for the Young’s modulus of the surface; *γ* stands for the surface energy density.

On the basis of the Griffith equation, *A* is assumed to be a constant. Hence, *F*$\sqrt{A\pi }$ is a function of *Eγ*. Here, *F*$\sqrt{A\pi }$ denotes a separating force (the force required to separate the fouling organism from the surface), and a low Young’s modulus (*E*) leads to a low separating force (*F*$\sqrt{A\pi }$). The low Young’s modulus is beneficial for fouling organisms to isolate from the surface due to low separating force. The experimental results in the present study also are in accord with the fracture mechanics. The harmonic motion effect sheds new light on the effects of Young’s modulus on antifouling behavior. 

## 4. Conclusions

SR composite membranes containing 0.36 wt % GO exhibited excellent antifouling performance in this study, while they are low-cost with inexpensive silicone rubber as the main material. Hence, these membranes may be promising for applications in antifouling. The combined effects of color and Young’s modulus determine the antifouling performance of GOSR membranes. The findings provide insight that could facilitate the fabrication of new antifouling coatings; specifically, lighter colors and low Young’s moduli provide superior performance. In addition, we provide a novel insight into the role of the elastic modulus toward the antifouling performance. 

## Supplementary Materials

The following are available online at https://www.mdpi.com/1996-1944/12/16/2608/s1, Figure S1: SEM images of diatoms (Triceratium sp.) on silicon slice after culturing for 2 days, Figure S2: Specimens were fixed to a hexagonal prism, Figure S3a: SEM image of GO nanosheets, Figure S3b: TEM image of GO nanosheets, Figure S3c: Raman spectra of GO nanosheets, Figure S3d: FTIR spectra of GO nanosheets, Figure S4: Colors of the membranes with different GO contents, and Figure S5: Roughness or GOSR membranes with different GO contents, Table S1: Nomenclature and values, Table S2: Elemental compositions of membranes.
