# Supplementary material for: Combined Effects of Color and Elastic Modulus on Antifouling Performance: A Study of Graphene Oxide/Silicone Rubber Composite Membranes
Source: Materials (Basel). 2019 Aug 16;12(16):2608. doi: 10.3390/ma12162608 (PMC6720792; doi:10.3390/ma12162608)
Supplement: Supplementary file 1 [file materials-12-02608-s001.pdf]

materials

Supplementary Materials

Combined Effects of Color and Elastic Modulus on Antifouling Performance: A Study of Graphene Oxide/Silicone Rubber Composite Membranes

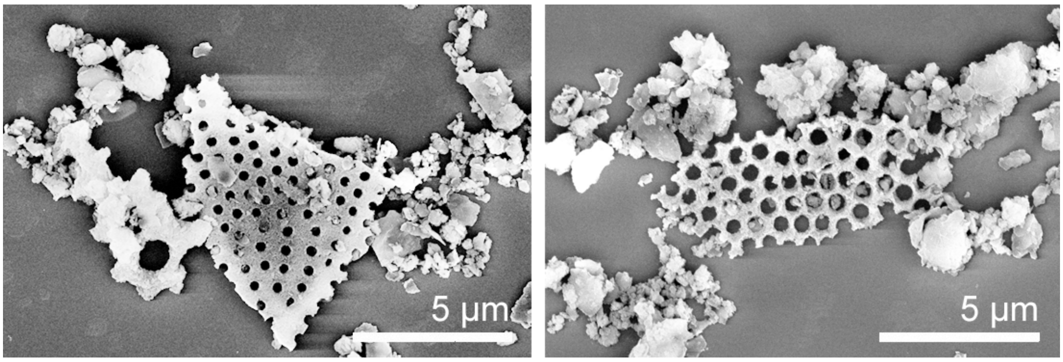

Figure S1. SEM images of diatoms (*Triceratium* sp.) on silicon slice after culturing for 2 days.

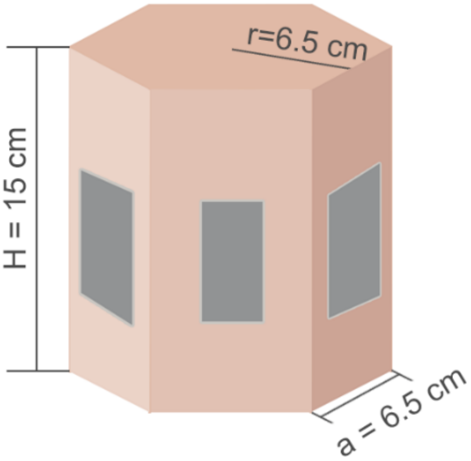

Figure S2. Specimens were fixed to a hexagonal prism.

The linear velocity (V) near the specimens is determined by $V = 2 \times \pi \times r \times \omega$, where ω is the rotation speed. In our test, ω = 500 rad/min. Therefore, V = 3.4 m/s.

The Reynolds number (*Re*) is an important dimensionless quantity in fluid mechanics that helps help predict the flow patterns in different fluid flow situations. *Re* is determined by the following equation:

$$Re = \frac{\rho V d_H}{\mu} \quad (1)$$

where

$$d_H = \frac{2ab}{a+b} \tag{2}$$

Table S1. Nomenclature and values.

Nomenclature		Value
ρ	water density	0.997 g/cm^3 (25 °C)
V	rate of water flow	3.4 m/s
d_H	hydraulic diameter of rectangular tube (fully filled)	
μ	dynamic viscosity	0.00089 Ns/m^2 (25 °C)
a, *b*	width and height of the section of the tube in test area	a = 6.5 cm and b = 15 cm

The results show that $Re = 3.45 \times 10^5$, which is much bigger than 4000; hence, there is turbulent flow in the test area, namely it is a turbulence generator.

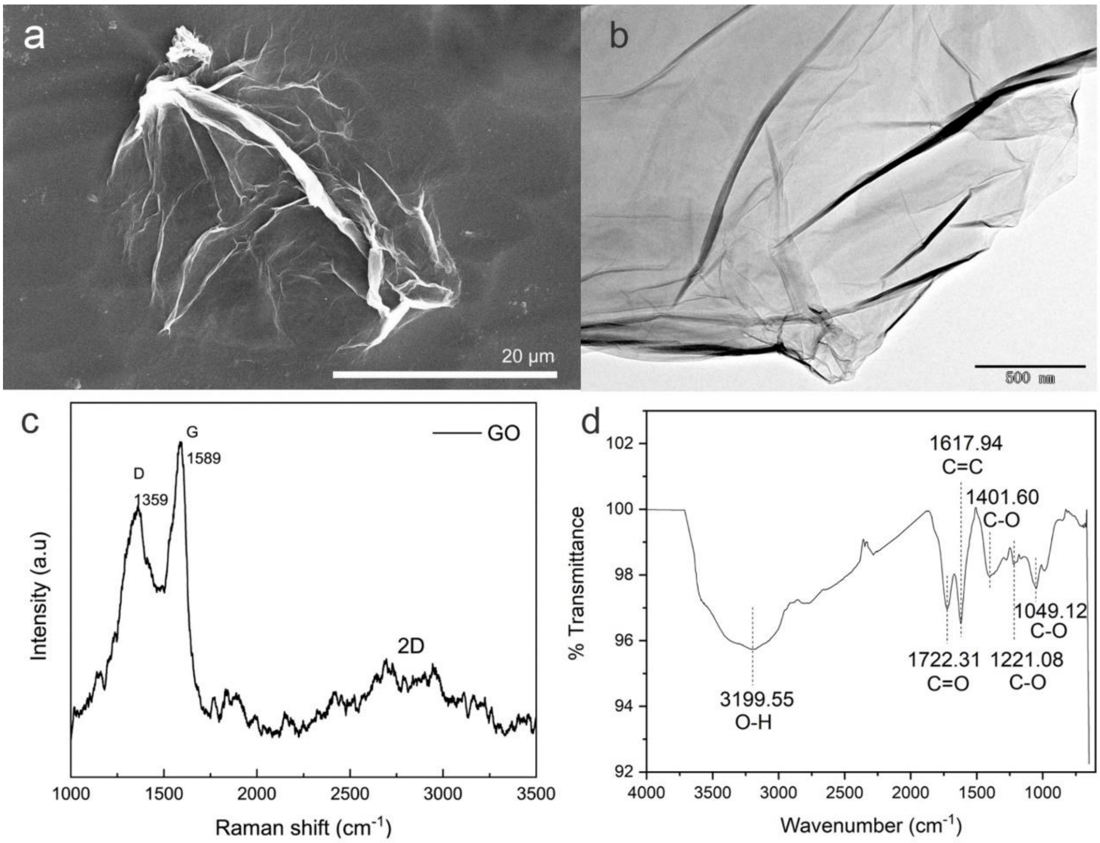

Figure S3. (**a**) SEM image of GO nanosheets, (**b**) TEM image of GO nanosheets, (**c**) Raman spectra of GO nanosheets, (**d**) FTIR spectra of GO nanosheets.

Table S2. Elemental compositions of membranes.

Elements/Specimens	0	0.16	0.36	0.64	1.28	2.56
C wt %	30.02	47.99	57.88	58.02	65.45	80.43
O wt %	33.43	38.21	32.11	36.16	32.66	18.94
Si wt %	36.55	13.8	10.02	5.81	1.89	0.63

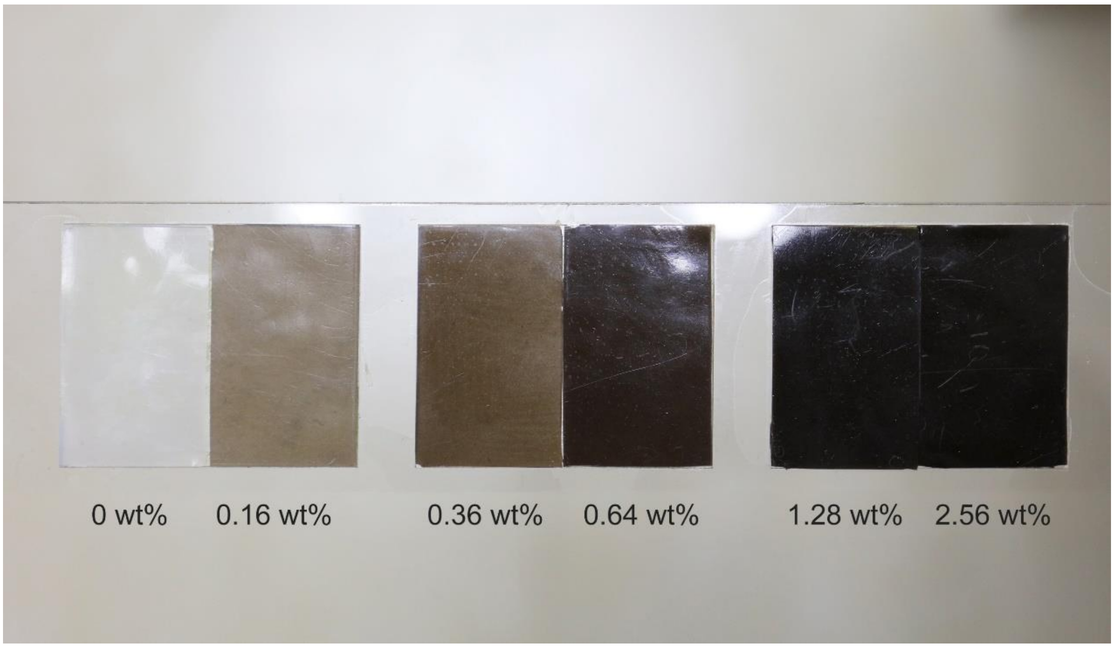

Figure S4. Colors of the membranes with different GO contents.

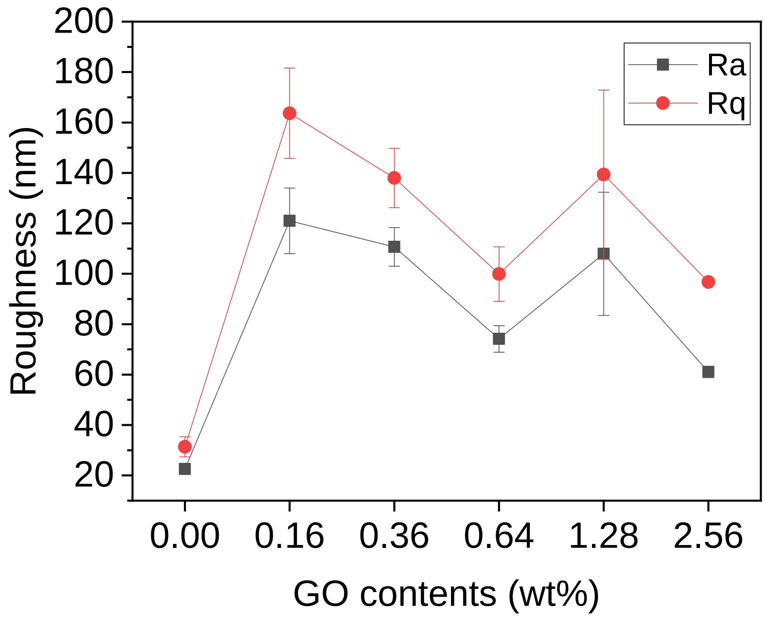

Figure S5. Roughness or GOSR membranes with different GO contents.

© 2019 by the authors. Submitted for possible open access publication under the terms and conditions of the Creative Commons Attribution (CC BY) license (http://creativecommons.org/licenses/by/4.0/).
